# Current state of neuroprotective therapy using antibiotics in human traumatic brain injury and animal models

**DOI:** 10.1186/s12868-024-00851-6

**Published:** 2024-02-29

**Authors:** Katharina Ritter, Pawit Somnuke, Lingjiao Hu, Eva-Verena Griemert, Michael K.E. Schäfer

**Affiliations:** 1 Department of Anesthesiology, University Medical Center, Johannes Gutenberg-University Mainz, Langenbeckstraße 1 (Bld. 505), Mainz, 55131 Germany; 2 Department of Anesthesiology, Faculty of Medicine Siriraj Hospital, Mahidol University, Bangkok, 10700 Thailand; 3 Department of Gastroenterology, Nanxishan Hospital of Guangxi Zhuang Autonomous Region, Guilin, China; 4 Focus Program Translational Neurosciences (FTN, Johannes Gutenberg-University Mainz, Mainz, Germany; 5 Research Center for Immunotherapy, University Medical Center, Johannes Gutenberg- University Mainz, Mainz, Germany

**Keywords:** Traumatic brain injury, Neuroprotection, Neuroinflammation, Infection, Inflammation, Antibiotics, Minocycline, Doxycycline, Ceftriaxone, Trovafloxacin

## Abstract

TBI is a leading cause of death and disability in young people and older adults worldwide. There is no gold standard treatment for TBI besides surgical interventions and symptomatic relief. Post-injury infections, such as lower respiratory tract and surgical site infections or meningitis are frequent complications following TBI. Whether the use of preventive and/or symptomatic antibiotic therapy improves patient mortality and outcome is an ongoing matter of debate. In contrast, results from animal models of TBI suggest translational perspectives and support the hypothesis that antibiotics, independent of their anti-microbial activity, alleviate secondary injury and improve neurological outcomes. These beneficial effects were largely attributed to the inhibition of neuroinflammation and neuronal cell death. In this review, we briefly outline current treatment options, including antibiotic therapy, for patients with TBI. We then summarize the therapeutic effects of the most commonly tested antibiotics in TBI animal models, highlight studies identifying molecular targets of antibiotics, and discuss similarities and differences in their mechanistic modes of action.

## Introduction

Traumatic brain injury (TBI) is a major cause of death and disability worldwide. Sixty-nine million individuals worldwide are estimated to sustain a TBI each year [1] and survivors are often left with enduring symptoms that can significantly affect their quality of life [2]. There are significant socio-economic consequences for TBI patients [3] and high medical costs associated with TBI [4, 5]. In Europe, TBI occurs most commonly due to falls, which has overtaken traffic accidents as the leading cause of injury in recent years [6, 7]. A bimodal age pattern (under 14 years old and older than 65 years) has been reported [8]. Young children aged 0–4 years are particularly vulnerable, with over 50,000 hospitalizations recorded annually in the US [9]. Due to the different mechanisms causing TBI, sex and age are unequally distributed. While falls are more common in older women, traffic accidents, violence and sports injuries are the predominant causes of TBI in young men. The overall proportion of men reported to suffer a TBI is always higher than that of woman [10], but functional outcomes do not differ significantly between men and woman [11]. However, women may be more susceptible to persistent cognitive symptoms after mild TBI than men [12].

The pathophysiology of TBI is multifactorial and the primary injury triggers a complex cascade of events, including impaired cerebral autoregulation of perfusion, blood-brain barrier (BBB) leakage, edema formation, oxidative stress, disruption of Ca^2+^ homeostasis, and mitochondrial dysfunction [13–16]. These interconnected processes lead to neuronal cell death and long-lasting brain inflammatory responses that can aggravate a wide range of clinical conditions [17] and can also have significant effects on peripheral organs [18]. However, it has become clear that inflammation has both harmful but also beneficial effects on neuronal survival and brain repair, e.g. by promoting the clearance of cell debris [17, 19–22]. Recent findings also link dysfunction of the glymphatic-meningeal lymphatic system with secondary events after TBI, such as edema and increased intracranial pressure (ICP), brain inflammatory responses, and insufficient clearance of cell debris and toxic metabolites [23–26].

Given the great heterogeneity in etiology, presentation, pathophysiology, complexity, and outcome, the care for patients with TBI proves to be a challenge [27]. In fact, the complexity of TBI pathophysiology limits the possibility for a general or causal therapeutic approach and the unpredictability of the individual pathophysiology requires close monitoring of the injured brain to tailor the treatment to the patient`s specific status [14].

Antibiotics are often part of the treatment of TBI patients. They can prevent or cure infections that can occur in open head injury patients, after neurosurgical interventions, or in mechanically ventilated patients (see studies listed in Table 1). Antibiotics can also have therapeutic effects independent of their anti-microbial activity, as shown by studies in TBI animal models (see studies listed in Table 2). In this review, we briefly summarize the current treatment options and clinical use of antibiotics in TBI. We further provide an overview of the therapeutic effects of the most commonly tested antibiotics in TBI animal models, highlight studies identifying molecular targets of antibiotics, and discuss similarities and differences in their mechanistic modes of action.

**Table 1:** Selected studies reporting treatment effects of antibiotics in human TBI

	Reference	Subjects and survival/follow up	Treatment groups, dosage and route	Treatment effects
Minocycline (MINO)	Minocycline reduces chronic microglial activation after brain trauma but increases neurodegeneration [57]	Cross-sectional study of patients with ≥ 6 mths after moderate to severe TBI without surgery, age range 20–65 years, ~ 80% male, follow up at 12 wk and 6 mths	1. Healthy controls (*n* = 64) 2. TBI, no MINO (*n* = 5) 3. TBI + MINO (*n* = 9) oral (100 mg), twice daily for 12 wk, all patients underwent arterial plasma sampling for NFL to evaluate neurodegeneration,^11^C-PBR28 PET to determine microglial activation, MRI to determine structural data	1. Reduced chronic microglial activation 2. Increased plasma levels of NFL
	Effects of minocycline on neurological outcomes in patients with acute traumatic brain injury: a pilot study [58]	Prospective randomized study of moderate to severe TBI patients undergoing surgery within 12 h after admission, age range 42.5 ± 15.8 years with 88.2% male, follow up at 6 mths	1. TBI + placebo (*n* = 20) 2. TBI + MINO (*n* = 14) oral (100 mg), oral twice daily, first dose within 24 h after admission then continued 2 times/day for 7 d	1. Reduced serum levels of NSE at 5d after admission 2. Improvement of GCS values from 1 d to 5 d after admission 3. No difference of 6-mths survival
Doxycycline (DOX)	The effect of doxycycline on neuron-specific enolase in patients with traumatic brain injury: a randomized controlled trial [59]	Randomized-controlled trial of moderate (GCS: 9–12) and severe TBI (GCS: 3–8) patients admitted < 24 h, age range 18–70 years with 50% male, follow up until 28 d after discharge	1. TBI + placebo (*n* = 20) 2. TBI + DOX (*n* = 20), oral (100 mg), twice daily for 7d	1. Reduced serum levels of NSE at 3 d and 7 d after admission 2. Increased GCS value at 7 d and at discharge 3. No difference in length of stay, number of deaths and mean survival days
Vancomycin and Meropenem	Efficacy and safety of intrathecal meropenem and vancomycin in the treatment of postoperative intracranial infection in patients with severe traumatic brain injury [60]	Retrospective analysis of patients with intracranial infection after severe TBI and surgical intervention (craniotomy), age range 30 ± 9 years, 53.5% male, all patients survived until the end of the study (6 mths)	1. Control group (*n* = 43), vancomycin (1 g) and meropenem (2 g), i.v. administration for 2 wk every 12 h, and meropenem every 8 h 2. Experimental group (*n* = 43), vancomycin (20 mg) and meropenem 20 mg, intrathecal administration for two weeks, once daily and meropenem twice daily	Intrathecal administration of antibiotics resulted in 1. Improved response rate (reduced intracranial infection) 2. Faster cure time in experimental group 3. Reduced treatment costs 4. Lower incidence of adverse effects
combined antibiotics	Effects of antibiotic prophylaxis on ventilator-associated pneumonia in severe traumatic brain injury. A post hoc analysis of two trials [54]	Retrospective analysis using two databases collected from 25 ICUs, age range 23–52 years with 85% male, follow up until 28 d after discharge	1. Control group, no antibiotic prophylaxis (*n* = 149) 2. Antibiotic prophylaxis (*n* = 146), 93% (*n* = 136) i.v. within 2 d after TBI, 72% of the patients received penicillins (mostly amoxicillin-clavulanate), 23% cephalosporins (mostly 1st or 2nd generation), 4% aminoglycosides, 1% 3rd generation cephalosporins and 0.3% metronidazole	1. Antibiotic prophylaxis reduced the occurrence and early incidence of ventilator-associated pneumonia 2. Mortality was not affected
	Early antibiotic administration is independently associated with improved survival in traumatic brain Injury [53]	Retrospective study on TBI patients admitted to the ICU, age range 59.7 ± 23 years with 65% male, most patients presented with blunt head trauma, recruitment of patients who survived longer than 48 h after admission	1. EARLY group: Patients with i.v. administration within 48 h after admission including cefazolin, gentamicin or vancomycin followed by additional antibiotics e.g. penicillins, cephalosporins, macrolides, aminoglycosides, fluoroquinolones or tetracyclines (*n* = 189) 2. Non-EARLY group: Patients who received antibiotics later than 48 h (*n* = 299, antibiotics not specified)	1. EARLY patients were younger than non-EARLY (54.2 ± 22.9 vs. 61.5 ± 22.2 years) 2. EARLY group presented with hypotension, lower GCS values, longer hospital and ICU stay and lower risk of mortality 3. Administration of early antibiotics independently correlated with lower mortality
	Antibiotic prophylaxis in penetrating traumatic brain injury: analysis of a single-center series and systematic review of the literature [61]	1. Retrospective single-center study, age range 32 ± 13 years, 20 male and 1 female, follow-up until 31 d after admission. 2. Systematic review of 14 studies including patients with penetrating head injury (total *n* = 327, sex not specified)	Single-center study: 1. Control, no prophylactic antibiotics 2. Cefazolin monotherapy 3. Various regimens of broad-spectrum antibiotics including vancomycin, ceftriaxone, and metronidazole, i.v. first dose within 24 h after admission, the continued up to 30 d Systematic review: 1. No prophylactic antibiotics 2. Single or combination of antibiotic regimen, i.v. at various time points ranging from single injection intra-operatively to repetitive injections for 3–7 d	Single-center study: Reduced numbers of patients with CNS infection after antibiotic prophylaxis (12%) vs. control (75%). Systematic review: (1) Among all patients from 14 studies, 66% received prophylaxis (2) The proportion of CNS infection in patients with and without prophylaxis was 17% and 19%, respectively 3. Short course of prophylactic antibiotics is recommended; i.v. cefazolin or ceftriaxone every 12 h + metronidazole every 6 h if organic debris is present in the wound

Abbreviations: GCS = Glasgow Coma Scale, ICU = intensive care unit, MINO = Minocycline, MRI = magnetic resonance imaging, NFL = Neurofilament light chain, NSE = neuron-specific enolase, PET = positron emission tomography

**Table 2:** Selected studies providing evidence for neuroprotective effects of antibiotics in rodent models of TBI

	Reference	Injury model, subjects, and survival time	Treatment groups, sample size, drug administration	Antibiotics treatment effects
Ceftriaxone	Ceftriaxone treatment preserves cortical inhibitory interneuron function via transient salvage of GLT-1 in a rat traumatic brain injury model [72].	LFP model of TBI, Sprague-Dawley male rats, 12 wk-old, survival 1 wk and 6 wk	1. TBI-saline 2. TBI-ceftriaxone (250 mg/kg), i.p. 30 min after TBI, then every 24 h for 7 d, *n* = 5–7/group	1. Transiently increased GLT-1 protein and glutamate transporter gene (SLC1A2) expression 2. Prevention of cortical interneuron dysfunction
	Neuroprotective effect of ceftriaxone in a rat model of traumatic brain injury [73].	WD model of TBI Sprague-Dawley male rats, 10–12 wk-old, survival 1 d, 3 d, and 5 d	1. sham-saline, *n* = 30 2. TBI-saline, *n* = 60 3. TBI-ceftriaxone (200 mg/kg), *n* = 60, i.p. immediately after TBI, then every 24 h for 5 d	1. Attenuated brain edema 2. Improved spatial learning and cognitive function 3. Increased hippocampal GLT-1 protein expression 4. Reduced neuronal autophagy in the hippocampus
	The beta-lactam antibiotic, ceftriaxone, provides neuroprotective potential via anti-excitotoxicity and anti-inflammation response in a rat model of traumatic brain injury [74].	WD model of TBI, Sprague-Dawley male rats, 180–220 g in size, survival 1 d, 2 d, 3 d, and 7 d	1. Sham-saline, *n* = 18 2. TBI-saline, *n* = 27 3. TBI-ceftriaxone (200 mg/kg), *n* = 27, i.v. after TBI (single dose)	1. Reduced spatial learning and memory deficits at 7 d after TBI 2. Attenuated cerebral edema at 1–3 d after TBI 3. Reduced levels of IL-1β, IFN-ɣ, and TNF-α expression in brain tissue at 1–3 d after TBI 4. Transient up-regulation of GLT-1 in brain tissue at 48 h
	Ceftriaxone treatment after traumatic brain injury restores expression of the glutamate transporter, GLT-1, reduces regional gliosis, and reduces post-traumatic seizures in the rat [75].	LFP model of TBI, Long-Evans male rats, 8–9 wk-old, survival 7 d	1. Sham-saline 2. TBI-saline 3. TBI-ceftriaxone (200 mg/kg), i.p., first dose at 30 min after TBI then every 24 h for 7 d, *n* = 7/group	1. Preservation of GLT-1 protein expression at 7 d after TBI 2. Attenuated astrogliosis in brain tissue (anti-GFAP) 3. Reduction in length and frequency of post TBI seizures
Trovafloxacib	Trovafloxacin attenuates neuro-inflammation and improves outcome after traumatic brain injury in mice [76].	Controlled cortical impact (CCI), C57BL/6 male mice, 10 wk-old, survival 1 d and 6 d	1. Sham 2. TBI-saline 3. TBI-trovafloxacin (60 mg/kg), i.p. at 1 h, 24 h, and 48 h after CCI, *n* = 8/group	1. Improved locomotor recovery 2. Reduced levels of MMP9 and SBDPs protein from brain tissue 3. Attenuated BBB leakage 4. Reduced hematoma size 5. Partially reduced mRNA levels of pro-inflammatory cytokines from brain tissue 6. Attenuated mRNA expression of neuroinflammatory markers MPO, GFAP, Iba1, CD68
Minocycline (MINO)	Minocycline Attenuates High Mobility Group Box 1 Translocation, Microglial Activation, and Thalamic Neurodegeneration after Traumatic Brain Injury in Post-Natal Day 17 Rats [77].	CCI model, Sprague-Dawley male rats, postnatal day 17, survival 7 and 14 d	1. Sham (naïve) 2. CCI-saline 3. CCI-MINO (90 mg/kg) i.p., first dose at 10 min and second dose at 20 h, *n* = 3–11/group	1. Reduced expression of the damage biomarker HMGB1 in the brain after 24 h 2. Reduced number of microglia (anti-Iba1) 3. Reduced neuronal cell death (Fluoro-Jade) 4. Inconclusive effects on motor function 5. Slightly improved spatial memory determined (Morris Water Maze)
	Sex Differences in Thermal, Stress, and Inflammatory Responses to Minocycline Administration in Rats with Traumatic Brain Injury [78]	CCI model, male and female rats, 8–10 wk-old, survival 35 d	1. Sham-saline 2. Sham-MINO (50 mg/kg) 3. TBI-saline 4. TBI-MINO (50 mg/kg), i.p., first dose 1 h after CCI, then once daily for 3 d, *n* = 14–16/group	1. Suppressed restraint stress-induced increase of plasma corticosterone 2. Inhibited CCI-induced hyperthermia 3. Increased IL-6 and IL-1β levels in the hippocampus of female rats but not in male rats
	Acute minocycline administration reduces brain injury and improves long-term functional outcomes after delayed hypoxemia following traumatic brain injury [79]	CCI model with delayed hypoxemia, male and female C57BL/6J mice, 8 wk-old, survival 7 d and 6 mths	1. sham 2. TBI-saline 3. TBI-MINO, i.p, 45 mg/kg, 90 mg/kg, 180 mg/kg in saline (dose finding study), first dose 22–25 h after CCI, repeated twice at 2 d and 3 d after CCI (pre-clinical study), *n* = 13–15/group	1. 180 mg/kg MINO resulted in 80% mortality at 3 d after CCI, 45 mg/kg was ineffective 2. 90 mg/kg followed by 5 × 45 mg/kg MINO reduced hippocampal microglia activation and neurodegeneration 7 d after CCI 3. 90 mg/kg followed by 5 × 45 mg/kg MINO improved long-term fear memory responses and synapse density 6 mths after CCI No sex-specific differences were reported
Minocycline + N-acetylcyteine (MINO + NAC)	Minocycline plus N-acetylcysteine protect oligodendrocytes when first dosed 12 h after closed head injury in mice [80].	Closed head injury (CHI), C57BL/6 male mice, 15–17 wk-old, survival 2 d, 4 d, 7 d, and 14 d	1. Sham-saline 2. TBI-saline 3. TBI-NAC (75 mM) 4. TBI-MINO (22.5 mM) 5. TBI-MINO + NAC (22.5 mM + 75 mM), i.p. at 12 h, 24 h, and 48 h after CCI, no sample size provided	CHI-MINO + NAC resulted in; 1. Preservation of oligodendrocytes in the corpus callosum 2. Attenuated loss of CNPase and PLP in the corpus callosum
	Minocycline plus N-Acetylcysteine Reduce Behavioral Deficits and Improve Histology with a Clinically Useful Time Window [81].	CHI, C57BL/6 male mice, 15–17 wk-old, CCI model, male rats (strain and age not specified), survival 14 d	1. Sham-saline 2. TBI-saline 3. TBI-NAC (75 mM) 4. TBI-MINO (22.5 mM) 5. TBI MINO + NAC (22.5 mM + NAC 75 mM), i.p., first dose at 6 h, 12 h, or 24 h after CCI and the second and third dose at 2 d and 3 d after CHI, respectively, *n* = 4–11/group	1. Improvement in spatial navigation, learning and memory (Barnes Maze, place avoidance test) 2. Improved preservation of neurons 3. improved preservation of myelin in the corpus callosum
Doxycycline (DOX)	Doxycycline alleviates acute traumatic brain injury by suppressing neuroinflammation and apoptosis in a mouse model [82].	Weight-drop model of TBI, Swiss Albino male mice, 25–35 g, survival 72 h	1. Sham-Saline 2. TBI-Saline 3. TBI-low dose DOX (10 mg/kg) 4. TBI-high dose DOX (100 mg/kg), i.p., first dose 30 min after TBI then every 8 h for 6 more doses, *n* = 5/group	1. Dose-dependent reduction in brain edema and hemorrhage 2. Reduced expression of the T cell marker CD3 in brain tissue 3. Reduced number of microglia (anti-Iba-1) 4. Attenuated expression of the proinflammatory cytokine IL-6 in brain tissue 5. Attenuated neuronal and glial cell death (TUNEL assay)
	Doxycycline improves traumatic brain injury outcomes in a murine survival model [83]	CCI model, C57BL/6J mice, survival 6 d	1. sham 2. TBI 3. TBI + DOX (20 mg/kg), i.v., first dose 2 h after CCI and then every 12 h until 6 d after CCI, *n* = 10–15/group	DOX improved neurological outcome, wire grip and ataxia scores
	Doxycycline prevents blood-brain barrier dysfunction and microvascular hyperpermeability after traumatic brain injury [84]	CCI model, C57BL/6 mice (18–25 g), intravital microscopy at 10–70 min after TBI, *n* = 5–7/group	1. Sham 2. TBI 3. TBI + DOX (20 mg/kg), i.v. 10 min after TBI	1. DOX decreased BBB hyperpermeability 2. DOX inhibited MMP-9 enzyme activity

Abbreviations: CCI = controlled cortical impact, CHI = closed head injury, CD = Cluster of differentiation, CNPase = cyclic nucleotide phosphodiesterase, DOX = Doxycycline, GFAP = glial fibrillary acidic protein, HMGB1 = High-mobility group box 1, Iba-1 = Ionized calcium-binding adaptor molecule 1, IL = Interleukin, IFN-ɣ = Interferon-gamma, i.p. = intraperitoneal, i.v. = intravenous, LFP = lateral fluid percussion, MINO = Minocycline, MMP-9 = Matrix metalloproteinase 9, MPO = Myeloperoxidase, NAC = N-Acetyl-Cysteine, PLP = proteolipid protein, SBDPs = spectrin breakdown products, TNF-α = Tumor necrosis factor alpha, TUNEL = TdT-mediated dUTP-biotin nick end labeling, WD = weight-drop

### Current treatment options in human patients with TBI

Acute treatment options for patients with TBI encompass surgical interventions, such as evacuation of intracranial hematomas, repair of skull fractures, decompressive craniectomy, and supportive measures to relieve symptoms and maintain homeostasis.

In recent years, significant advances have been made in neuromonitoring, neuroimaging, and surgical interventions to maintain normocapnia, normoxia and normothermia, and to positively influence outcome [28–30]. However, surgical interventions are often ambiguous or fail to improve outcome with regard to surgical technique (e.g. craniotomy versus decompressive craniectomy) and timing [31–35]. Advances in neuromonitoring comprise, for example, the detailed analysis of ICP using machine learning models, which can improve the prediction of outcomes and mortality [36–40] It is likely that novel and improved neuromonitoring methods will have an increasing impact on treatment strategies [36, 41, 42].

It is important to note that rigorous treatment trials (e.g., randomized control trials) or meta-analyses are complicated by the absence of a standardized methodological approach and focus on individualized treatment strategies with frequent re-evaluation. Indeed, this could be a major reason for the failure of most pharmaceutical interventions in clinical trials. Nevertheless, some trials have opened up new therapeutic options for other diseases or expanded the possibilities for the application of neuroprotective drugs [43].

### Antibiotic therapy in human patients with TBI

Post-injury infections are common in-hospital complications of TBI, affecting up to 55% of patients with moderate-severe TBI [18] and up to 75% of adult patients with severe TBI [44, 45]. The high susceptibility to infection has been associated with general immune suppression in TBI patients [44, 46]. The major infection types are those affecting the lower respiratory tract and surgical site [44], including nosocomial pneumonia after aspiration, device-related infections (e.g. ventilator-associated pneumonia or catheter-related infections), and meningitis following cranial procedures (e.g. surgery and/or device placement) [48–50]. A negative association between post-injury infections, clinical outcome or higher mortality rates was reported in TBI [51, 52]. Antibiotic treatment has been postulated to be relevant to the outcome of TBI patients [43] and early administration of antibiotics in TBI patients results in lower mortality rates [53]. Along this line, early antibiotic prophylaxis delayed, and prevented nosocomial infections and ventilation-associated pneumonia in patients with severe TBI but did not change length of hospital stay or mortality [54, 55]. Positive clinical effects, albeit not reaching a statistically significant level, were reported in a meta-analysis on antibiotic prophylaxis for ventricular drain placement in patients with basal skull fractures [56]. To the best of our knowledge, there is currently no general evidence-based guideline for the administration of antibiotics in TBI patients.

Currently, a wide range of antibiotics is used for anti-infectious treatment in TBI patients. The choice of antibiotics may depend not only on the patient`s health status and the diversity of pathogenic bacteria classes infecting the patient, but also on hospital-specific guidelines and physician-specific preferences. Nevertheless, some recommendations and anti-infective regimens have been published for antibiotic treatment of patients with open or penetrating TBI. The predominating strategy is early intravenous administration of antibiotics that cover the spectrum of gram-positive and gram-negative bacteria (e.g. amoxicillin/clavulanic acid, or 2nd or 3rd generation cephalosporins, such as cefazolin, ceftriaxone, or cefuroxime), optionally supplemented with an aminoglycoside and vancomycin, and metronidazole or meropenem if anaerobic infection is suspected [50, 61–65]. To our knowledge, there are no direct studies on the sex-specific effects of antibiotics and no sex-specific recommendations regarding the type of antibiotics.

It should be noted that prolonged prophylactic antibiotic use (> 48 h) for nosocomial pneumonia in trauma patients has been reported to carry the risk of antibiotic complications and a higher incidence of multidrug-resistant bacteria [66]. Another possible caveat is that antibiotic therapy has a significant impact on the gut microbiome and can promote opportunistic pathogenic bacteria, such as *Clostridioides difficile*, potentially associated with severe symptoms in patients [67–70]. Colonization with multi-drug resistant bacteria was found in fecal samples from 25 of 39 TBI patients (64%) as early as 48 h after admission to an intensive care unit [71]. This supports the hypothesis that TBI associates with an increased ratio of pathogenic to commensal bacteria, as suggested in a recent systematic review that included an observatory study with human TBI patients and six animal studies [85]. Such a microbiome shift has also been observed in chronic TBI, potentially leading to long-lasting changes in bacterial nutrient utilization, which may contribute to reduced availability of amino acids in TBI patients [86, 87].

Altogether, infections are relevant complications in TBI patients and require symptomatic antibiotic therapy, whereas prophylactic antibiotic therapy appears to be rather empirical at present and possible negative effects, such as antibiotic-induced microbiome dysbiosis, must be taken into account. As the role of the gut-brain axis in TBI is beyond the scope of this review article, we would like to refer readers to excellent reviews that have focused on this exciting topic [88–91].

### Antibiotics in animal models of TBI

In contrast to the small number of clinical studies, a larger number of experimental studies have examined the effects of different classes of antibiotics in animal models of TBI. Details of selected studies are shown in Table 2.

The models used comprise the controlled cortical impact (CCI) model, the lateral fluid percussion (LFP) model, and closed-head injury weight-drop models. It should be kept in mind that none of these injury models alone can model the wide spectrum of human TBI and that rodent models are dominating the research field [92, 93]. However, rodent models can shed light on pathophysiologically relevant processes also known to occur in human TBI patients. Importantly, they provide the possibility to perform standardized preclinical studies, thereby preventing uncontrolled experimental variabilities, which may add to the already highly heterogeneous etiology, presentation, and complexity of human TBI. However, it should be noted that most experimental studies used male mice and sex was not considered as a biological variable. This would require comparing male and female mice in the same study. Briefly summarized, experimental data on minocycline administered alone, or in combination with N-acetylcysteine, support the inhibition of microglia activation, associated neuroinflammation, and anti-apoptotic effects as the principal mechanism of action [78–81]. Two experimental TBI studies considered sex as a biological variable [78, 79]. Taylor et al. reported short-term anti-pyretic effects of minocycline irrespective of sex and increased IL-1β and IL-6 levels in the hippocampus of minocycline-treated ovariectomized female rats, but not in male rats [78]. A comprehensive study by Celorrio et al. showed inhibition of injury-induced microglia activation and neurodegeneration in the hippocampus as well as long-term improved fear memory performance and increased synapse densities following minocycline treatment without significant sex-differences [79]. Doxycycline also showed anti-inflammatory and anti-apoptotic effects [83], BBB-protective effects [84], and improved outcomes [83, 94]. Ceftriaxone was found be neuroprotective, which has been associated with the up-regulation of the glutamate transporter GLT-1 (also termed EAAT2, encoded by the SLC1A2 gene) [72, 94–96]. Trovafloxacin, showed anti-inflammatory and neuroprotective actions attributed to the inhibition of the pannexin channel Panx1 [76].

Consistent with observations in TBI patients, several animal studies provided evidence that TBI induces rapid and long-lasting changes in gut microbiota and metabolites, which in turn may influence TBI pathogenesis [71, 86, 97–103]. For example, gut microbiome dysbiosis has been associated with impaired neurogenesis and increased pro-inflammatory activation of microglia in a mouse model of TBI [102, 103] and alterations in the gut microbiome were found to influence the permeability of the BBB [104], which is a key structure in TBI pathogenesis. Adverse effects of bacterial metabolites produced under pathogenic conditions may account for these observations [89, 105]. These results from animal studies support the above-mentioned safety considerations for the use of antibiotics in human TBI patients with regard to possible adverse effects related to changes in the microbiome.

In the following sections, the effects and mechanisms of commonly used antibiotics in rodent models of TBI are reviewed and discussed in light of existing data on human TBI.

### Minocycline

Minocycline, a tetracycline derivative, is considered the most effective at providing neuroprotection [106–108]. There is a substantial number of therapeutic studies in animal models of TBI that demonstrate its neuroprotective effects (examples in Table 2) [77, 79, 81, 109–118], including synergistic effects with N-acetylcysteine [80, 119–122]. The experimental data support the inhibition of microglia activation and associated neuroinflammation as the principal mechanism of action. Several properties of minocycline may contribute to these effects. In general, tetracyclines including minocycline form poorly soluble chelates with bivalent and trivalent cations, particularly calcium, magnesium, aluminium, and iron [123]. This property may be related to the anti-oxidative and neuroprotective effects of minocycline. Indeed, biochemical in vitro data support anti-oxidative actions of minocycline, showing a Fe^2+^- and Fe^3+^- chelating activity [124] and a scavenger function for peroxynitrite [125]. Minocycline also attenuated iron neurotoxicity in cultures of primary mouse neurons [126] and after experimental intracerebral hemorrhage [127]. In addition, minocycline may mitigate the iron-induced pro-inflammatory activation of microglia and macrophages following the uptake of erythrocytes, hemoglobin, and heme after CNS injury [128].

Other work suggested that minocycline inhibits the release of cytochrome c from mitochondria, a key step during the early phase of apoptosis [129], which may contribute to neuroprotective effects independent from anti-inflammatory actions [130]. Finally, indirect evidence in a murine closed-head trauma model suggested that cannabinoid receptors are required for neuroprotective actions of minocycline because protective actions of minocycline were prevented by cannabinoid receptor antagonists [131].

Collectively, the evidence for minocycline can explain a wide spectrum of treatment effects. The positive pleiotropic effects in experimental models as well as its safety profile and effective crossing of the BBB suggest that minocycline is particularly suitable for the treatment of patients after TBI [132]. However, the results of some experimental TBI studies do not support sustained neuroprotection or improved behavioral outcomes after treatment with minocycline [133–136].

Nevertheless, the promising results from preclinical studies prompted first clinical trials, however the results on the neuroprotective benefits of minocycline remain controversial [57, 58, 137, 138]. It has been concluded that minocycline shows potential efficacy in treating acute but not chronic TBI [121]. In fact, minocycline administration for 12 weeks caused reduction of microgliosis in chronic TBI patients. However, this effect coincided with increased plasma levels of neurofilament light chain (NF-L), a biomarker for axonal damage, which has led to the conclusion that minocycline increased neurodegeneration [57]. The authors discussed that chronically activated microglia may have trophic rather than deleterious effects, consistent with earlier results in rhesus monkey [139, 140]. In support of this explanation, Safaiyan et al. identified a population of white-matter associated microglia with a potentially protective role during white matter aging and disease [141]. An alternative, albeit speculative, explanation would be that increased plasma levels of NF-L were caused by minocycline-mediated reduction of microglia with high phagocytic activity and not by neurodegeneration.

However, up until now, most of the human trials could be considered pilot studies. Prospective, randomized multicenter trials, particularly those focusing on optimal dosing and timing, are needed [142]. Moreover, human clinical trials investigating the potentially synergistic combination of minocycline with N-acetylcysteine are warranted [143].

### Doxycycline

Doxycycline is another antibiotic of the tetracycline class that has been investigated in TBI animal models (Table 2). In general, tetracyclines such as doxycycline as well as minocycline show iron-chelating activity [123].

Recent studies showed that doxycycline attenuates BBB leakage and vascular hyperpermeability [84], suppresses neuroinflammation and apoptosis [82], and improves neurological outcomes [83, 94]. Similar to minocycline, the iron-chelating effects of doxycycline may contribute to its neuroprotective effects, as excess iron can damage the injured brain via ferroptosis or other iron-mediated mechanisms [144, 145]. Indeed, doxycycline protects neurons from iron-dependent ferroptosis in vitro [146].

Important molecular insights were gained by combining *in silico* molecular docking studies and enzymatic activity assays in brain tissue preparations after murine TBI. Evidence was provided that doxycycline binds to the active site of the proteolytic enzyme matrix metalloproteinase 9 (MMP-9) [84] and decreases MMP-9 serum levels after experimental TBI in rats [147]. These results have potential clinical relevance because MMP-9 is associated with BBB disruption and secondary hemorrhage [148] and may serve as an outcome-relevant CSF biomarker along with other MMPs in TBI patients [149]. The underlying molecular mechanisms are diverse due to the broad substrate specificity of MMP-9, as demonstrated by secretome analysis in murine experimental autoimmune encephalomyelitis, which uncovered 119 potential MMP-9 substrates [150]. Interestingly, experimental stroke studies using gene-deficient mice provided indirect evidence that MMP-9 is also a possible target of minocycline [151], and minocycline also exhibits iron-chelating activity similar to doxycycline [123], suggesting common mechanisms of action.

Thus, doxycycline may interfere with iron-mediated deleterious processes and inhibit MMP-9, a relevant pathophysiological molecular target. Given clinical findings of reduced serum levels of the neuronal injury marker NSE after doxycycline treatment [59], rigorous testing of doxycycline in prospective clinical trials would be important.

### Ceftriaxone

Ceftriaxone is a β-lactam antibiotic of the cephalosporin group. Neuroprotective effects of ceftriaxone were shown after experimental TBI in rats (Table 2). Posttraumatic administration of ceftriaxone reduced brain edema, pro-inflammatory cytokine expression, and cognitive deficits. Moreover, restored protein expression levels of the glutamate transporter GLT-1 (also termed EAAT2, encoded by the SLC1A2 gene) were associated with the neuroprotective effects of ceftriaxone [74]. Subsequent studies confirmed the association between ceftriaxone, GLT-1, and neuroprotection [94–96]. Moreover, repeated administration of ceftriaxone attenuated neuronal apoptosis [96, 152] and neuronal autophagy [95], and TBI-induced astrogliosis and seizures [75]. The findings also suggested that ceftriaxone-mediated restoration of GLT-1 expression attenuates glutamate excitotoxicity. In support of this hypothesis, it was demonstrated that ceftriaxone protects cortical parvalbumin-positive interneurons susceptible from excitotoxicity, thereby attenuating TBI-induced loss of intracortical inhibition [72]. More recent data demonstrated that administration of ceftriaxone during the chronic phase of experimental TBI reduces seizure susceptibility to the convulsant pentylenetetrazol, possibly due to augmented expression levels of the glutamate transporters GLT-1 and GLAST- 1 (also termed EAAT1, encoded by the SCL1A3 gene) [153].

There is evidence for a molecular target of ceftriaxone. Given the crucial role of the nuclear factor kappa-B (NF-kB) signaling pathway for GLT-1/EAAT2 induction by ceftriaxone [152, 154], a recent study addressed the question of how ceftriaxone triggers transcriptional activity of NF-kB without increasing pro-inflammatory gene expression. Using *in silico* 3D homology modelling, a possible explanation was provided by predicting ceftriaxone binding sites and its conformational effects on NF-kB´s site-specific DNA interaction responsible for EAAT-2 expression [155]. Some questions remain about the precise molecular mechanisms and molecular targets of ceftriaxone. The view that extracellular glutamate is an exclusive trigger of excitotoxicity is not without controversy [156–158], and glutamate biosensor imaging studies indicated that ceftriaxone decreased glutamate release independent of GLT-1 expression levels [159].

Altogether, experimental data support neuroprotective effects of ceftriaxone. The underlying mechanisms are still elusive, but some evidence point to a crucial role of the transcription factor NF-kB and the release and uptake regulation of the excitatory neurotransmitter glutamate.

### Trovafloxacin

Trovafloxacin is a broad-spectrum fluoroquinolone-derived antibiotic. Treatment studies using a murine CCI model of TBI showed that trovafloxacin has neuroprotective and anti-inflammatory effects [76]. On the other hand, structural similarities of fluoroquinolones to endogenous glutamate receptor ligands, such as kynurenic acid, suggest an interaction with ligand-gated glutamate receptors. Indeed, trovafloxacin, along with other fluoroquinolones, evoked excitatory responses in acute hippocampal slice preparations from rats [160]. Moreover, trovafloxacin has been found to be associated with adverse CNS events [161]. Later studies identified trovafloxacin as an inhibitor of pannexin-1 (Panx-1) channels [162]. These channels mediate paracrine and autocrine signaling via ions and small metabolites including Ca^2+^ and ATP [163], thereby serving as sensors and effectors for changes in the cellular microenvironment [164]. Panx-1-mediated ATP release has been associated with pathogenic processes in TBI, such as inflammasome activation and pyroptosis, immune cell infiltration as well as glia proliferation and scar formation [165, 166]. Panx-1 is expressed in blood cells, immune cells, neurons and glia (www.proteinatlas.org). However, subjecting myeloid-specific Panx-1 knockout mice to the CCI model of TBI revealed that Panx-1 mediates the infiltration of peripheral immune cells, leukocytes, macrophages, and neutrophils, whereas the number of brain-resident microglia was not affected by myeloid-specific Panx-1-deficiency. Interestingly, both MMP-9 levels and spectrin breakdown products (SBDPs), a marker of axonal injury and excitotoxicity in the CCI model of TBI [167], were attenuated in myeloid-specific Panx-1 knockout mice [168].

Overall, several studies indicate potent effects of trovafloxacin in the context of neuronal excitation as well as brain inflammation. It remains to be clarified whether these effects are equally relevant after TBI and whether they are synergistic or opposing in terms of neuroprotective and anti-inflammatory actions.

### Other antibiotics

This review focuses on the most commonly investigated antibiotics in experimental TBI. There are only a few studies on other antibiotics in brain injury models. For example, ampicillin has been shown to increase the expression of the glutamate transporter GLT-1 and to reduce neuronal damage in a rodent model of transient forebrain ischemia [169]. This suggests a similar mode of action to ceftriaxone, but data on experimental TBI are lacking. Ampicillin has also been combined with metronidazole, neomycin and vancomycin to achieve depletion of gut microbiota in experimental TBI [170]. However, this study did not aim to reveal neuroprotective effects of antibiotics independent of their anti-microbial activity. Vancomycin, a bactericide glycopeptide antibiotic used primarily for the spectrum of gram-positive infections, mediated BBB-related neuroprotection in a *Staphylococcus epidermis*-potentiated model of neonatal hypoxic-ischemic brain injury. However, these observations may be due to vancomycin`s anti-infective rather than neuroprotective effect [171]. Rifampicin, an effective antibiotic for the treatment of tuberculosis, showed neuroprotective effects and improved cognitive impairment after global cerebral ischemia in rats, which has been attributed to the activation of the pathophysiologically relevant transcription factor NRF2 [172]. Rifampicin further inhibits neuronal cell death via inhibition of microglia activation in vitro [173]. It has been proposed to reduce neurotoxicity of amyloid beta protein through an antioxidative mechanism [174], and therefore could be a promising test candidate for experimental TBI. In summary, some data on the antibiotics ampicillin, vancomycin, and rifampicin suggest neuroprotective potential and further studies are needed to test their potential clinical relevance.

## Conclusions

There is an unmet need for clinical therapeutic options in the treatment of TBI. Increasing evidence suggests that antibiotic therapies may have neuroprotective and anti-inflammatory effects besides their anti-microbial mechanisms of action. However, the evidence for prophylactic antibiotic therapy is somewhat empirical at present and evidence-based standard operating procedures for the prophylactic use of antibiotics need to be established. The benefit of antibiotic therapy should be carefully considered in the light of adverse effects on the gut microbiome and the gut-brain axis. Some evidence from experimental research points to a mechanistic overlap of neuroprotective and anti-inflammatory actions of antibiotics. At least between minocycline and doxycycline, there are mechanistic similarities in terms of antioxidant and iron-chelating activities, and molecular targets, such as MMP-9. Further identification and characterization of molecular targets is of paramount importance to demonstrate the relevance of standardized antibiotic-based therapies in a variety of animal models of TBI across different laboratories.

## Data Availability

Not applicable.
